# Illness perceptions, social support and antiretroviral medication adherence in people living with HIV in the greater Accra region, Ghana

**DOI:** 10.1002/nop2.797

**Published:** 2021-02-24

**Authors:** Nella O. Anakwa, Enoch Teye‐Kwadjo, Irene A. Kretchy

**Affiliations:** ^1^ Department of Psychology School of Social Sciences University of Ghana Legon Ghana; ^2^ Department of Pharmacy Practice and Clinical Pharmacy School of Pharmacy University of Ghana Legon Ghana

**Keywords:** antiretroviral therapy, HIV, illness perceptions, medication adherence

## Abstract

**Aim:**

To assess how illness perceptions and social support influence antiretroviral medication adherence in a HIV patient population in Ghana.

**Design:**

This study used a correlational research design with cross‐sectional data.

**Method:**

A total of 235 people living with HIV at two general hospitals in the Greater Accra Region of Ghana provided data on illness perceptions, social support and medication adherence. Hierarchical multiple regression test was used to analyse the data.

**Results:**

Illness perceptions' facets of timeline, personal control and treatment control were negatively associated with medication adherence, whereas emotional response was positively associated with adherence. Further, significant other support was negatively associated with adherence. Family and friend support were not associated with adherence. The findings offer preliminary evidence that illness perceptions may have utility for medication adherence in a HIV patient population in Ghana.

## 1 INTRODUCTION

Adherence to antiretroviral medication is crucial for the management of HIV/AIDS (Langebeek et al., 2014; Sweileh, 2018). For example, optimal HIV medication adherence brings about viral suppression and delays in disease progression as well as the prevention of resistance, disability and death (Egger et al., 2002; Wood, 2004). Conversely, poor adherence to antiretroviral medication is known to lead to poor health outcomes in people living with HIV (PLHIV) and to increased healthcare costs (Al‐Dakkak et al., 2013; Cutler et al., 2018; Munakata et al., 2006). HIV remains a major health problem in Ghana (Ghana Aids Commission, 2018; Teye‐Kwadjo, 2014; UNAIDS, 2018). As of the year 2017, the total HIV population in Ghana was estimated to be 313,063. Of this number, approximately 113,171 adults and children were receiving antiretroviral therapy (ART), with about 30,410 newly diagnosed adults and children preparing to initiate ART (Ghana Aids Commission, 2018; UNAIDS, 2018). Ghana has adopted the UNAIDS’ 90–90–90 goals, which aim to make 90% of all people who are living with HIV to know their status, put 90% of those diagnosed with HIV infection on sustained ART and attain a 90% viral suppression amongst those receiving ART by the year 2020 (UNAIDS, 2014).

## 2 BACKGROUND

Within the broader framework of ending the HIV epidemic by the year 2030, Ghana is implementing the “treat‐all” strategy (Nash et al., 2018), which requires that every PLHIV in Ghana be provided with ART. Whilst it is important to place PLHIV on ART, research into adherence to ART ought to be seen as a critical component of the “treat‐all” policy. In addition, to improve adherence to ART and to achieve 90% viral suppression success, it is crucial to identify variables that predict adherence (DiMatteo et al., 2012). However, there is little information on adherence to ART amongst PLHIV in Ghana (for exception, see Anokye‐Kumatia et al. 2018; Nichols et al., 2019). Anokye‐Kumatia et al. (2018) investigated adherence to ART in a sample of perinatally infected HIV adolescents in Ghana. They found that adherence to ART was low and that forgetfulness and inability to go for medication refills were the key determinants of low adherence in their sample (see also Anakwa et al., 2020; Nichols et al., 2019). Viewed from a health promotion perspective, more research that informs our understanding of adherence to ART in Ghana seems warranted, as this research can help identify factors associated with adherence to ART. The factors identified can then be targeted for intervention within the scope of the “treat‐all” policy.

According to the World Health Organization ([WHO], 2003), adherence reflects the extent to which a person's medication‐taking behaviour matches the recommendations of a healthcare provider. Recent work has categorized factors influencing medication adherence into patient‐related factors, health system‐related factors, medication‐related factors and caregiver‐related factors (Ammon et al., 2018). The factors could be categorized further into facilitators and barriers (see Ammon et al., 2018). The present research focuses on patient‐related factors. Two important patient‐related factors found in meta‐analyses to predict adherence to ART are illness perceptions or illness beliefs (Leone et al., 2016; Pala & Steca, 2015) and social support (Langebeek et al., 2014; Malta et al., 2010).

Illness perceptions arise from the common‐sense model of illness representations proposed by Leventhal et al. (1980). This model holds that patients (e.g. PLHIV), through cognitive and affective processes, create mental representations of their illness experiences (Cameron & Leventhal, 2003). In other words, the model suggests that patients use information available to them regarding their illness to help make sense of their illness by creating mental representations of their illness.

According to the common‐sense model of illness representations (see Farmer, 2012; Hagger & Orbell, 2003), there are five components of cognitive illness perceptions/ representations. They are (a) *consequences* (beliefs regarding the impact of the illness on the patient), (b) *timeline* (beliefs about the chronicity of the illness), (c) *personal control* (beliefs about the uncontrollability of the illness by the patient), (d) *treatment control* (beliefs about incurability of the illness through treatment and medication) and (e) *identity* (beliefs about the illness’ symptoms as the patient experiences them). Two additional components represent emotional illness perceptions/representations. They are (f) *concern* (fears and worries the patient experiences about the illness) and (g) *emotions* (the negative emotional arousal the illness brings about in the patient). One component assesses illness comprehensibility. This component is (h) *understanding* (beliefs about the manifestations and treatment of the illness), and a final component of the model assesses (i) *causal representation* of the illness by means of an open‐ended statement, which requires the patient to list the three most important causes of their illness (see Cameron & Leventhal, 2003; Leventhal et al., 2003).

The common‐sense model of illness perceptions/representations has been used successfully to predict medication and treatment adherence in various patient populations, including diabetes (Nie et al., 2018; Owiredua et al., 2018), hypertension (Chen et al., 2011) and cancer populations (Llewellyn et al., 2007). For example, Leone et al. (2016) examined the association of illness perceptions with virologic success amongst patients who were HIV‐positive in Italy. They found that perceived consequences of illness and emotional perceptions of illness were related to virologic success. The authors suggested that illness perceptions should be used in future interventions to promote adherence to ART.

Moreover, in a study to examine the associations between illness perceptions and post‐traumatic growth amongst 225 newly diagnosed HIV‐positive men who have sex with men in China, Lau et al. (2018) found that the cognitive illness representation subscales of timeline, consequence and identity were negatively associated with post‐traumatic growth whereas personal control, treatment control and understanding were positively associated with post‐traumatic growth. Growing research indicates that illness perceptions have a substantial influence on treatment and medication adherence (see Wu et al., 2018). Despite this, to our knowledge, no study has investigated the relationship between illness perceptions and adherence to ART in the Greater Accra Region of Ghana. Therefore, in accordance with the reviewed literature, we proposed the following research questions (RQ) and hypotheses (H).

**RQ1**: How do cognitive illness perceptions, emotional illness perceptions and illness comprehensibility relate to HIV medication adherence?

**RQ2**: How does social support relate to HIV medication adherence?

Illness perception facets of timeline, consequence, concern, identity, personal control and treatment control (i.e. cognitive illness representations) would have a negative relationship with medication adherence.

Illness perception facets of emotional response and concern (i.e. emotional illness representations) would have a positive relationship with medication adherence.

Illness perception facet of understanding (i.e. illness comprehensibility) would have a positive relationship with medication adherence.

According to Gottlieb and Coppard (2009), social support describes the social, psychological and interpersonal assistance that optimizes health and well‐being of individuals. In other words, social support reflects the tangible (e.g. money) and intangible (e.g. motivation) that is offered to a person by their social network. Social support has been found to exert a positive influence on the mental health of persons with HIV by acting as a protective factor against adverse health outcomes (DiMatteo, 2004; Wohl et al., 2010). Social support comprises three important agents, which are the family, friends and significant others (Zimet et al., 1988). In a systematic review amongst HIV‐infected drug users, Malta et al. (2010) found better ART adherence outcomes amongst drug users who reported receiving social support than drug users who did not receive social support. Similarly, in a meta‐analysis involving 207 studies to identify the predictors of adherence to combination ART, Langebeek et al. (2014) found that social support was strongly positively associated with adherence. In accordance with Langebeek et al. (2014) and Malta et al. (2010), we proposed the following hypothesis.

Social support would have a positive relationship with medication adherence. That is, PLHIV with higher levels of perceived social support from family, friends and from significant others would report greater medication adherence.

### 2.1 Aim

The ways HIV patients make sense of their own illness as well as the social support available to them can help determine their commitment to treatment and medication adherence. Therefore, the aim of this study was to explore the relationship between illness perceptions, social support and adherence to ART in PLHIV in Ghana. Another aim was to identify facets of illness perceptions and social support that show promise for effective medication adherence.

### 2.2 Design

This study used a correlational research design with cross‐sectional data.

## 3 METHOD

### 3.1 Participants and procedure

Participants were people living with HIV (*N* = 235; *n* = 115, men; *n* = 120, women) who were receiving antiretroviral therapy at two general hospitals in the Greater Accra Region of Ghana. Participants’ age ranged from 18 to 64 years with a mean age of 35.94 (*SD* = 10.26). Participants were recruited in April and May 2019 using purposive sampling techniques. Of a purposive sample of 240 participants recruited for this study, 235 provided the data for the current analysis. Five people abstained due to disinterest and conflict with timing for the data collection. Of the total HIV population in Ghana in 2017 (i.e. 313,063), the Greater Accra Region had the highest estimated HIV population of 73,556, representing 23.5% (Ghana AIDS Commission, 2018). The two general hospitals were selected because of their known supportive care services for people living with HIV/AIDS. Participants completed informed consent forms prior to the completion of a paper‐and‐pencil questionnaire battery on medication adherence, illness perceptions and social support in English language. They also completed a biographical questionnaire. For most of the participants who were highly literate in the English language, the study questionnaires were handed to them in an envelope to complete for collection later by the research team. The research team assisted those who needed help in completing the questionnaire. Confidentiality of participation and anonymity of questionnaire responses were ensured. Participants were made aware that they could withdraw from the study at any time without suffering any consequences. Ethics requirements did not permit us to compensate the respondents for their time.

### 3.2 Measures

#### 3.2.1 Demographic information

Information on participant's age, gender, educational level, marital status and religious affiliation, employment status and treatment duration was obtained and included in the analysis as control variables.

#### 3.2.2 Medication adherence

Antiretroviral medication adherence was assessed by the 6‐item Simplified Medication Adherence Questionnaire (SMAQ) developed and validated by Knobel et al. (2002). This scale was developed to assess adherence in people on antiretroviral treatment in clinical settings. The original scale asks six questions regarding adherence and requires respondents to provide a “yes” or “no” response to the questions. Knobel et al. (2002) reported a Cronbach's alpha of 0.75 for the original scale. In this study, we modified the items slightly from a question format to a statement format and used a 5‐point Likert response scale ranging from, 1 (*very untrue of me*) to 5 (*very true of me*). The reason for the modification was to have it measured on the same metric (i.e. Likert response format) as those of our independent variables to facilitate the use of parametric tests. Item number six (#6) on the original scale was not used in this study because it was considered redundant as it elicits a similar response to that of item number four (Knobel et al., 2002). The items as validated and used in this study are as follows; “I ever forgot to take my HIV medication.”, “I am careless at times about taking my HIV medication.”, “If I feel worse at times, I stop taking my HIV medication.”, “Over the past weekend, I did not take any of my HIV medication.” and “Over the last week, I sometimes did not take my HIV medication.” We used exploratory factor analysis procedures to examine the scale's construct dimensionality and validity. Scale scores were summed, and total scores were calculated such that higher scores indicated medication non‐adherence. Cronbach's alpha coefficient for the five items used in this study was 0.77, 95% confidence interval (CI) [.72, .81].

#### 3.2.3 Illness perceptions

We used a modified version of the 9‐item Brief Illness Perception Questionnaire (B‐IPQ) developed and validated by Broadbent et al. (2006) to assess illness perceptions. The B‐IPQ assesses patients’ cognitive and affective illness perceptions/representations towards a health condition (e.g. HIV), using nine single‐item subscales with different response scales. The first eight items on the B‐IPQ are rated on an 11‐point scale, ranging from 0 to 10. Broadbent et al. (2006) reported a good test‐retest reliability between *r* = 0.71 and *r* = 0.72 for the subscales over a six‐week interval. The B‐IPQ has been shown to possess discriminant validity, as it can distinguish between different illnesses (Broadbent et al., 2006; Foot et al., 2019).

The following are the subscales and the items that indicate them; *consequences* (How much does your illness affect you?), *timeline* (How long do you think your illness will continue?), *personal control* (How much control do you feel you have over your illness?), *treatment control* (How much do you think your treatment can help your illness?), *identity* (How much do you experience symptoms from your illness?), *concern* (How concerned are you about your illness?), *understanding* (How well do you feel you understand your illness?), *emotional response* (How much does your illness affect you emotionally?) and *causal representation* (Please list in rank‐order the three most important factors that you believe caused your illness.). To maintain the negative and threatening view of illness perceptions inherent in the common‐sense model and in line with previous research (see Nur, 2018), the indicators of personal control, treatment control and understanding were reverse‐scored. In this study, scores were calculated separately for each subscale. High scores on each subscale reflect greater illness perceptions/beliefs about the negative consequences of HIV. Because the causal representation item provides for an open‐ended response (categorical data), it was analysed separately using descriptive statistics (see Table 1). In accordance with previous research and for psychometric reasons (see Haines et al., 2019; Nie et al., 2018), we did not compute an overall score for the B‐IPQ because each subscale is indicated by only one item. Cronbach's alpha was also not computed.

**TABLE 1:** Sociodemographic characteristics of participants (*N* = 235)

Variable	Frequency	Percentage	Mean	*SD*
Age			35.94	10.20
Treatment duration (months)			48.52	40.38
Gender				
Male	115	48.9		
Female	120	51.1		
Marital status				
Single	94	40.0		
Married	122	51.9		
Divorced	14	6.0		
Widowed	5	2.1		
Educational level				
Basic	50	21.3		
Secondary	104	44.3		
Tertiary	81	34.5		
Religious affiliation				
Christianity	160	68.1		
Muslim	70	29.8		
Other	5	2.1		
Employment status				
Unemployed	62	26.4		
Part time	73	31.1		
Full time	100	42.6		
What important factors do you believe caused your HIV?				
Hereditary factors	3	1.3		
Spiritual factors	24	10.2		
Unprotected sexual activity	154	65.5		
Unsterilized medical instruments	32	13.6		
Unknown	22	9.4		

#### 3.2.4 Perceived social support

Perceived social support was assessed by the 12‐item Multidimensional Scale of Perceived Social Support (MSPSS) developed by Zimet et al. (1988). The MSPSS is rated on a 7‐point Likert scale ranging from 1 (*very strongly disagree*) to 7 (*very strongly agree*). It is a widely used measure designed to assess participants’ perceptions of the sufficiency of the support they perceive or receive from family (e.g. “I get the emotional help and support I need from my family.”), friends (e.g. “My friends really try to help me.”) and significant others (e.g. “I have a special person who is a real source of comfort to me.”). The MSPSS has demonstrated good internal consistency reliability in a previous research in Ghana for family (0.86), friends (0.84), significant other (0.96) and social support total (0.86) in a population with albinism (Affram et al., 2019). In the present study, Cronbach's alpha coefficients were, family (0.89, 95% CI [.86, 0.91]), friends (0.81, 95% CI [.77, 0.85]), significant others (0.91, 95% CI [.89, 93]) and social support total (0.92).

### 3.3 Analysis

Data analyses were performed in the Statistical Package for the Social Sciences (SPSS, v21). Descriptive statistics on the sociodemographic variables were calculated (see Table 1). Normality checks, using the skewness criterion of −2 to +2 and kurtosis criterion of −7 to +7 as described by West et al. (1995), were performed on the main study variables. The study variables were normally distributed. Following this, descriptive statistics were computed. Next, internal consistency reliability was determined for each subscale with multi‐items, using Cronbach's coefficient alpha. Stratified alpha reliability (see Moussa, 2016) was computed for social support total. Then bivariate correlations were calculated between the main variables in the study. The correlation matrix is illustrated in Table 2. Hierarchical multiple regression was used to test the main hypotheses in this study, following the recommendation by Cohen et al. (2003). The analyses were conducted in a three‐step approach, based on the multidimensional nature of the variables in this study. The dependent variable was regressed on the independent variables in the analyses. At Step 1, the sociodemographic variables were entered into the regression equation as control variables. At Step 2, antiretroviral medication adherence was regressed on facets of illness perceptions. At Step 3, perceived social support was added to the model.

**TABLE 2:** Descriptive statistics and intercorrelations amongst the study variables (*N* = 235)

Variable		*M*	*SD*	1	2	3	4	5	6	7	8	9	10	11	12
1.	Adherence	14.92	4.45	—											
2.	Consequence	6.82	2.29	0.155^a^	—										
3.	Timeline	7.15	2.26	−0.056	0.275^c^	—									
4.	Personal	6.57	2.24	−0.395^c^	−0.302^c^	−0.236^c^	—								
5.	Treatment	8.03	1.99	−0.315^c^	−0.159^a^	−0.050	0.416^c^	—							
6.	Identity	5.03	2.56	0.195^b^	0.319^c^	−0.025	−0.180^b^	−0.400^c^	—						
7.	Concern	7.22	2.01	−0.018	0.279^c^	0.277^c^	−0.143^a^	0.046	0.066	—					
8.	Understanding	6.80	2.03	0.053	0.111	0.180^b^	0.119	0.177^b^	−0.123	0.059	—				
9.	Emotions	6.90	2.33	0.141^a^	0.385^c^	0.274^c^	−0.145^a^	−0.156^a^	0.238^c^	0.349^c^	−0.023	—			
10.	Family support	14.92	4.98	−0.153^a^	−0.099	−0.143^a^	0.319^c^	0.187^b^	−0.105	−0.151^a^	0.144^a^	−0.056	—		
11.	Friend support	12.08	4.56	−0.036	0.032	−0.296^c^	0.104	−0.022	−0.009	−0.167^a^	0.002	−0.125	0.225^b^	—	
12.	Significant other	14.38	5.61	−0.455^c^	−0.337^c^	−0.125	0.435^c^	0.277^c^	−0.205^b^	−0.078	−0.017	−0.156^a^	0.301^c^	0.251^c^	—

Abbreviations: *M*, mean; *SD*, standard deviation

^a^ *p* < .05

^b^ *p* < .01

^c^ *p* < .001

### 3.4 Ethics

Ethics approval and permission to conduct the study at the two hospitals were obtained from the University of Ghana (Ref#: ECH044/18‐19) and the Ghana Health Service (Ref#: GHS/GARHD/007/19). Informed consent was obtained from all of the participants in the study.

## 4 RESULTS

Descriptive statistics and responses to the open‐ended statement on the B‐IPQ, which asks participants to list the most important causal factors (i.e. causal illness representation dimension) of their HIV, are summarized in Table 1.

The bivariate correlations calculated between the study variables are illustrated in Table 2.

The results of the Hierarchical Regression analyses are presented in Table 3.

**TABLE 3:** Hierarchical linear regression of antiretroviral medication adherence on illness perceptions and perceived social support

	Antiretroviral medication adherence		
	R^2^	ΔR^2^	β
Step 1: Control variable	0.098	0.098^b^	
Age			−0.21^b^
Gender			−0.14^a^
Marital status			0.06
Education			0.00
Employment			0.06
Religious affiliation			0.17^b^
Treatment duration			−0.06
Step 2: Illness perception	0.299	0.201^c^	
Consequence			−0.04
Timeline			−0.18^b^
Personal control			−0.38^c^
Treatment control			−0.14^a^
Identity			0.05
Concern			−0.08
Understanding			0.12
Emotional response			0.17^b^
Step 3: Perceived social support	0.382	0.083^c^	
Family support			−0.03
Friend support			0.00
Significant other support			−0.33^c^

Note.

Gender (1 = male, 2 = female); Education (1 = basic, 2 = secondary, 3 = tertiary); Employment (1 = unemployed, 2 = part time, 3 = full time); Marital status (1 = single, 2 = married, 3 = divorced, 4 = widowed); Religion (1 = Christians, 2 = Muslims, 3 = Other)

^a^ *p* < .05

^b^ *p* < .01

^c^ *p* < .001

As can be seen in Table 3, the model at Step 1 was statistically significant, accounting for 9.8% variance in medication adherence. Specifically, the results showed that age (β = −0.21, *p* < .001) and gender (β = −0.14, *p* < .01) were negative significant predictors of medication adherence whereas religion (β = 0.17, *p* < .01) was a positive significant predictor. In addition, the model at Step 2 was statistically significant, with illness perceptions accounting for 20.1% variance in medication adherence (Δ*R^2^* = 0.201, *p* < .001). Specifically, results showed that cognitive illness perceptions facets of timeline (β = −0.18, *p* < .001), personal control (β = −0.38, *p* < .001) and treatment control (β = −0.14, *p* < .05) were each negatively significantly associated with medication adherence, providing a partial support for hypothesis one. Emotional illness perceptions facet of emotional response (β = 0.17, *p* < .01) was positively significantly associated with medication adherence, providing support for hypothesis two. However, illness perception facets of consequence, identity and concern were not associated with medication adherence in this sample (see Table 3). Further, illness comprehensibility (i.e. understanding; β = 0.12, *p > *.05) was not associated with medication adherence, disconfirming hypothesis three.

Moreover, the model at Step 3 was statistically significant, with perceived social support explaining additional 8.3% variance in medication adherence (ΔR^2^ = 0.083, *p* < .001). Specifically, the results showed that only support from significant others (β = −0.33, *p* < .001) was negatively significantly associated with medication adherence. Support from family and friends was not associated with medication adherence in this sample, disconfirming hypothesis four. In summary, the hierarchical model explained a total of 38.2% variance in medication adherence (see Table 3).

## 5 DISCUSSION

### 5.1 Illness perceptions and medication adherence

The aim of this study was to gain a better understanding of how illness perceptions and social support relate to antiretroviral medication adherence in PLHIV in Ghana. The results highlight the important ways in which illness perceptions and social support influence medication adherence in this sample. Consistent with our prediction, illness perceptions significantly influenced medication adherence. Specifically, the results showed that timeline (i.e. beliefs about the chronicity of HIV), personal control (i.e. beliefs about the uncontrollability of HIV through personal action) and treatment control (i.e. beliefs about treatment inefficacy and HIV incurability) were negatively associated with antiretroviral medication adherence in this sample. These results are consistent with a previous research amongst newly diagnosed HIV‐positive men who have sex with men, which reported high levels of negative illness perceptions between HIV and post‐traumatic growth (Wu et al., 2018).

In other words, patients living with HIV who perceived their illness as being more chronic were less likely to adhere to their antiretroviral medication. One possible explanation for this result is that patients’ beliefs about the chronicity of their condition (being long–term) may have served to discourage them from taking their antiretroviral medications. Another plausible reason is that the patients may be suffering from pill burden based on the continuous medication use. Similarly, patients who construed their illness as being more uncontrollable were also less likely to adhere to their antiretroviral medication.

Our result regarding the relationship between illness perceptions and medication adherence has the potential to help improve antiretroviral interventions in Ghana because it identifies patient‐related factors that could be amenable to change in interventions to improve adherence behaviour. A previous work has shown that negative illness perceptions such as beliefs about uncontrollability, incurability and chronicity of a health condition are associated with poor mental health outcomes (Petrie & Weinman, 2006). Similarly, Petrie et al. (2007) noted that illness perceptions influence illness management with important implications for mental health outcomes. Thus, our results highlight the importance of designing interventions to increase HIV patients’ personal control and treatment control in order to improve medication adherence. Extant literature has shown that the perception of control of a health condition is a crucial determinant of good mental health outcomes (Jessop & Rutter, 2003; Orbell et al., 2008).

Further, the results suggest that HIV patients who construed their HIV treatment as being highly unhelpful demonstrated low commitment to following their medication regimens. In other words, HIV patients’ beliefs about the inefficacy of their treatment and the incurability of the disease may have served to weaken their human agency in following their medication regimens. This result is consistent with findings by Gonzalez et al. (2007) which reported that some illness beliefs about adherence such as medication concerns, the necessity of taking medication and not trusting the medications were significant predictors of non‐adherence.

Conversely, emotional responses of patients to the HIV condition such as anger, sadness and guilt positively predicted medication adherence in this sample. This result shows that the higher the negative emotional arousal experienced by the patients in this study, the greater their commitment to medication adherence. This result corroborates that of earlier research amongst HIV‐positive patients by Leone et al. (2016) which reported that high‐negative emotions towards HIV positively predicted virologic success. In contrast to previous findings (see e.g. Lau et al., 2018; Leone et al., 2016), this study found no relationship between illness perception facets of consequence, identity, concern and understanding and medication adherence. That is, the current sample does not seem to consider those variables salient for medication adherence.

### 5.2 Perceived social support and medication adherence

Contrary to our prediction, perceived social support from family and friends was not related to medication adherence in this sample. This finding conflicts with the existing literature on social support from family and friends (Abramowitz et al., 2009; Asante, 2012). However, an interesting finding in this study is the negative association between perceived social support from significant others and medication adherence. That is patients with HIV who reported receiving strong support from significant others, were less likely to adhere to their medications.

A probable explanation is that whilst the efforts by significant others such as healthcare professionals, religious leaders and fellow HIV patients may be considered salient in HIV treatment, these individuals may not be readily available to prompt PLHIV to take their medications. In other words, the inverse relationship between significant other support and medication adherence may suggest that as support from significant others to PLHIV increases, adherence to HIV medication decreases because it is possible for PLHIV to reason that the support alone may be enough for them. This situation could be likened to risk compensation where PLHIV after receiving the necessary support from significant others, they rather begin to engage in riskier behaviour by not adhering to their medication regimen. For more information on risk compensation theory, see Lardelli‐Claret et al. (2003) and Thompson et al. (2002). This result is comparable to that of Ankrah et al. (2016) who found social support from healthcare providers (i.e. significant others) to be an important predictor of medication adherence amongst adolescents living with HIV in a teaching hospital in Ghana.

Ammon et al. (2018) reported similar results in a systematic review (see also Simoni et al., 2006). Finally, our independent variables together accounted for 38.2% of the variance in medication adherence. Although all cross‐sectional data are attended by measurement error, as the case may be in the present study, the variance explained represents a large effect size (Cohen's *f* ^2^ = 0.618). This practical significance seems to confirm the strength of the relationship between our independent variables and the dependent variable. Thus, HIV medication adherence interventions in Ghana that incorporate illness perceptions/representations and social support may prove to be effective.

## 6 LIMITATIONS

This study used self‐report measures to assess medication adherence, illness perceptions and perceived social support. Self‐report measures, despite their popularity, are known to be susceptible to response biases, which could mask true psychological traits or states. Another limitation to note is that illness perceptions were assessed with the Brief Illness Perception Questionnaire (B‐IPQ), which has nine subscales with one item indicating each subscale. Although it is well known that short measures can help to reduce respondent fatigue and research cost, we note that assessing a subscale with a single item might not be adequate to fully elicit the construct‐domains of a scale. Moreover, the analysis in this study is based on cross‐sectional data. These limitations should guide the interpretation of the results.

## 7 CONCLUSION

The results of this study extend the illness perceptions literature to the cultural context in Ghana. The study identified illness perception subscales of timeline, personal control, treatment control and emotional response to influence antiretroviral medication adherence, suggesting the utility of illness perceptions and by extension the common‐sense model in HIV medication adherence in Ghana. The findings imply that the beliefs that persons with HIV have about their illness could be considered as an important variable to include in adherence interventions. In addition, social support from significant others such as healthcare professionals negatively influenced medication adherence, suggesting that healthcare professionals in the HIV management value chain ought to take into consideration patient's views of the support they receive from them (health professionals) and how this could impact adherence. Taken together, the results highlight the importance of designing interventions to increase HIV patients’ personal control, treatment control, emotional response and to deconstruct beliefs about timeline in order to improve medication adherence in Ghana.

## CONFLICT OF INTEREST

The authors declare that they have no conflicts of interest to report.

## AUTHOR'S CONTRIBUTIONS

ETK, NOA and IAK conceptualized the study. NOA collected the data. ETK analysed the data and interpreted the results. ETK drafted the manuscript. NOA and IAK contributed to drafting. ETK and IAK reviewed the manuscript for critical intellectual content. All authors read and approved the final manuscript.

## Data Availability

The data on which the article reports are available from the corresponding author on reasonable written request.
